# Stroke burden attributable to high body mass index in China from 1990 to 2021 and projections to 2036 based on global burden of disease data

**DOI:** 10.1097/MD.0000000000049042

**Published:** 2026-05-29

**Authors:** Ye Tian, Wenbin Tian, Pei Zhang, Ning Yu, Chao Liu, Xuefang Liu, Huaihai Lu

**Affiliations:** a Department of Anesthesiology and Intensive Care, The Second Hospital of Hebei Medical University, Shijiazhuang, Hebei, China.

**Keywords:** body mass index, global burden of disease, stroke

## Abstract

The disease burden of stroke due to high body mass index (BMI) in China is unknown. This study investigated the burden from 1990 to 2021 and modeled projections for the next 15 years (2021–2036). Data from the Global Burden of Disease 2021 study were analyzed using join-point regression to determine trends in stroke burden attributable to high BMI in China and worldwide from 1990 to 2021. The autoregressive integrated moving average model was used to predict the burden from 2022 to 2036. The burden of stroke attributable to high BMI has been increasing in China since 1990, whereas the global trend shows a divergent trajectory. The burden associated with stroke varies among different stroke subtypes, gender, and age groups. In China, stroke mortality attributable to high BMI was projected to increase from 3.47 per 100,000 in 2021 to 4.80 per 100,000 in 2036. China’s public health authorities should focus on high-risk groups and ensure the implementation of a comprehensive “prevention-treatment-rehabilitation” intervention to reverse this trend.

## 1. Introduction

The global incidence of stroke has been steadily increasing over the past 3 decades, making stroke the second leading cause of mortality and third leading cause of disability worldwide, resulting in nearly 7 million deaths annually.^[1–4]^ Stroke incidence and mortality rates increase exponentially with age; individuals aged > 55 years account for nearly 75% of the total stroke burden.^[5–7]^ Recent reports indicate that the incidence and prevalence of stroke are higher in women than in men (6.4 vs 5.8 million and 56.4 vs 45.0 million, respectively).^[3]^ Notably, 90% of stroke-related fatalities and disability occur in low- and middle-income countries, where stroke incidence is increasing at an alarming rate.^[3]^

While metabolic syndrome confers an elevated risk for cerebrovascular disease, it is important to distinguish the independent contribution of high body mass index (BMI) as a modifiable risk factor for stroke. High BMI has been established as a distinct risk, with a dose–response association for both ischemic and hemorrhagic stroke (HS) incidence and mortality, confirmed in large-scale cohort analyses and epidemiological studies.^[8–13]^ According to the Global Burden of Disease Study 2021 (GBD 2021) methodology, which provides comprehensive, comparable data on global and national stroke burden, China faces a substantial and increasing burden of stroke attributable to high BMI.^[13]^

The age-standardized mortality rate (ASMR) associated with obesity is 63 cases per 100,000 individuals in China, and mortality rates have shown a consistent upward trend between 2000 and 2019, with a steeper increase among men.^[8]^ High BMI further leads to significant socioeconomic consequences, including health resource strain and productivity loss. Recent estimates suggest that the economic losses caused by high BMI-related stroke, as measured by disability-adjusted life years multiplied by gross domestic product per capita, represent a major and escalating financial burden for both families and the national health system.^[4,13]^

Despite growing awareness, China faces unique challenges in addressing high BMI and stroke risk compared with OECD countries. Differences in prevention and policy strategies, including implementation of the “Healthy China 2030” initiative, highlight the need for tailored and internationally comparable interventions. By quantifying the stroke burden and economic impact attributable to high BMI using the latest GBD 2021 data and aligning with global action frameworks, this study aims to provide robust scientific evidence for effective policy design and resource allocation in China.^[14,15]^

The purpose of this study is thus to clarify the burden and time trends of stroke attributable to high BMI in China, contextualize findings within global and national policy frameworks, and provide a scientific basis for targeted and cost-effective interventions.

## 2. Materials and methods

### 2.1. Data source

The data that support the findings of this study are openly available in the GBD 2021 public database at https://www.healthdata.org/research-analysis/about-gbd, reference number [Global Burden of Disease Collaborative Network. GBD 2021. Seattle, United States: Institute for Health Metrics and Evaluation, 2024]. The present study is a secondary analysis based on publicly available data.

The data were provided by the Institute for Health Metrics and Evaluation and quantified the health loss related to diseases, injuries, and risk factors across the world from 1990 to 2021. Attributable number, ASMR, deaths, years lived with disability, years of life lost, and disability-adjusted life years by selected risk factors were estimated by comparative risk assessment. The GBD study was approved by the University of Washington Institutional Review Board, and the deidentified, aggregated data were available with a waiver of informed consent.

The World Health Organization defines stroke as rapidly developing clinical signs of focal disturbance of cerebral function lasting more than 24 hours or leading to death, with no apparent cause other than that of vascular origin.^[11,16]^ In this study, stroke was categorized as ischemic stroke (IS) or HS (further classified as intracerebral hemorrhage [ICH] or subarachnoid hemorrhage [SAH]).^[17]^ IS encompasses all vascular events that limit blood flow to brain tissue, resulting in infarction, thromboembolic stroke, or atherosclerotic stroke, but excluding ICH. HS is defined as non-traumatic SH or ICH.

High BMI is defined as a BMI greater than a theoretical minimum risk level (20–25 kg/m^2^ in adults aged ≥ 20 years).^[18]^ Population exposure to high BMI was calculated for each country, age, sex, and year using a combination of spatiotemporal Gaussian process regression and mixed-effects models.^[19]^ High BMI was established as a risk factor for stroke by performing a systematic review of cohort studies and using causal criteria to examine the strength of the evidence. It should be noted that a negative lower bound for population-attributable fractions did not signify a protective effect but the absence of a relationship.

From GBD 2021, we obtained age-specific case and mortality rates as well as ASMR of stroke and its subtypes (IS, ICH, and SAH) attributable to high BMI in China between 1990 and 2021. The population for each age category and year was calculated as the number of deaths divided by the mortality rate. ASMR was standardized using the global age structure in 2021.

### 2.2. Join-point regression analysis

Join-point regression analysis was used to analyze the trend of stroke burden attributable to high BMI in China and worldwide from 1990 to 2021. The model builds a piecewise regression and performs trend fitting and optimization on the data points in each segment based on the temporal characteristics of the disease distribution.^[20]^ The model results were summarized using annual percent change (APC) and average annual percent change (AAPC). The APC was calculated as APC = [(*yx* + 1 − *yx*)/ *yx*] × 100% = (eβ1 − 1) × 100% to evaluate the trend of independent intervals of piecewise functions. The AAPC was calculated as AAPC = (e ∑ wiβi/∑wi − 1) × 100% to assess the average trend over the entire study interval. The analysis was conducted using Join-point Regression software v4.8.0.1 (April 2020; National Cancer Institute, Rockville). A *P* value <.05 was set as the threshold for statistical significance.

### 2.3. Autoregressive integrated moving average (ARIMA) model

During the autoregressive integrated moving average (ARIMA) modeling process, the differencing method was initially used to stabilize the time-series data. The auto.arima() function was used to select the best-optimized model based on the akaike information criterion (AIC). The underlying assumption was that data series are time-dependent random variables whose autocorrelation can be characterized by the ARIMA model, with future values predicted based on past values. The equation was expressed as *Yt* = ϕ1*Yt* − 1 + ϕ2*Yt* − 2 +... + ϕ*pYt* − *p* + *et* − θ1*et* − 1 −... − θ*qet* − *q*, where (ϕ1*Yt* − 1 + ϕ2*Yt* − 2 +... + ϕ*pYt* − *p* + *et*) is the autoregressive (AR) model; *et* − θ1*et* − 1 −... − θ*qet* − *q* is the moving average model; *Yt* − *p* is the observed value during the period (*t* − *p*); *p* and *q* are the model orders of AR and moving average, respectively; and et is the random error at the period of *t*.^[21]^ The time series in the ARIMA model was a stationary and stochastic sequence with zero mean. The ARIMA model was used to quantitatively describe future trends in stroke mortality attributable to high BMI from 2021 to 2036. Join-point segmentation logic diagrams were provided. Residual white noise was examined using diagnostic plots (ACF/PACF plots). ARIMA analysis and data visualization were performed using R v4.1 software (R Foundation for Statistical Computing, Vienna, Austria) using the “forecast,” “readxl,” “ggpubr,” and “ggplot2” packages.

## 3. Results

### 3.1. Trends in stroke burden attributable to high BMI, 1990 to 2021

ASMR of stroke attributable to high BMI showed an upward trend from 1990 to 2021 (Figs. S1 and S2). In China, the ASMR increased from 12.07 (95% confidence interval [CI]: 7.89–18.37) to 38.15 (95% CI: 32.22–44.93) per 100,000; globally, the increase was from 27.36 (95% CI: 13.56–42.88) to 47 (95% CI: 23.42–71.7) per 100,000.

**Figure 1.**
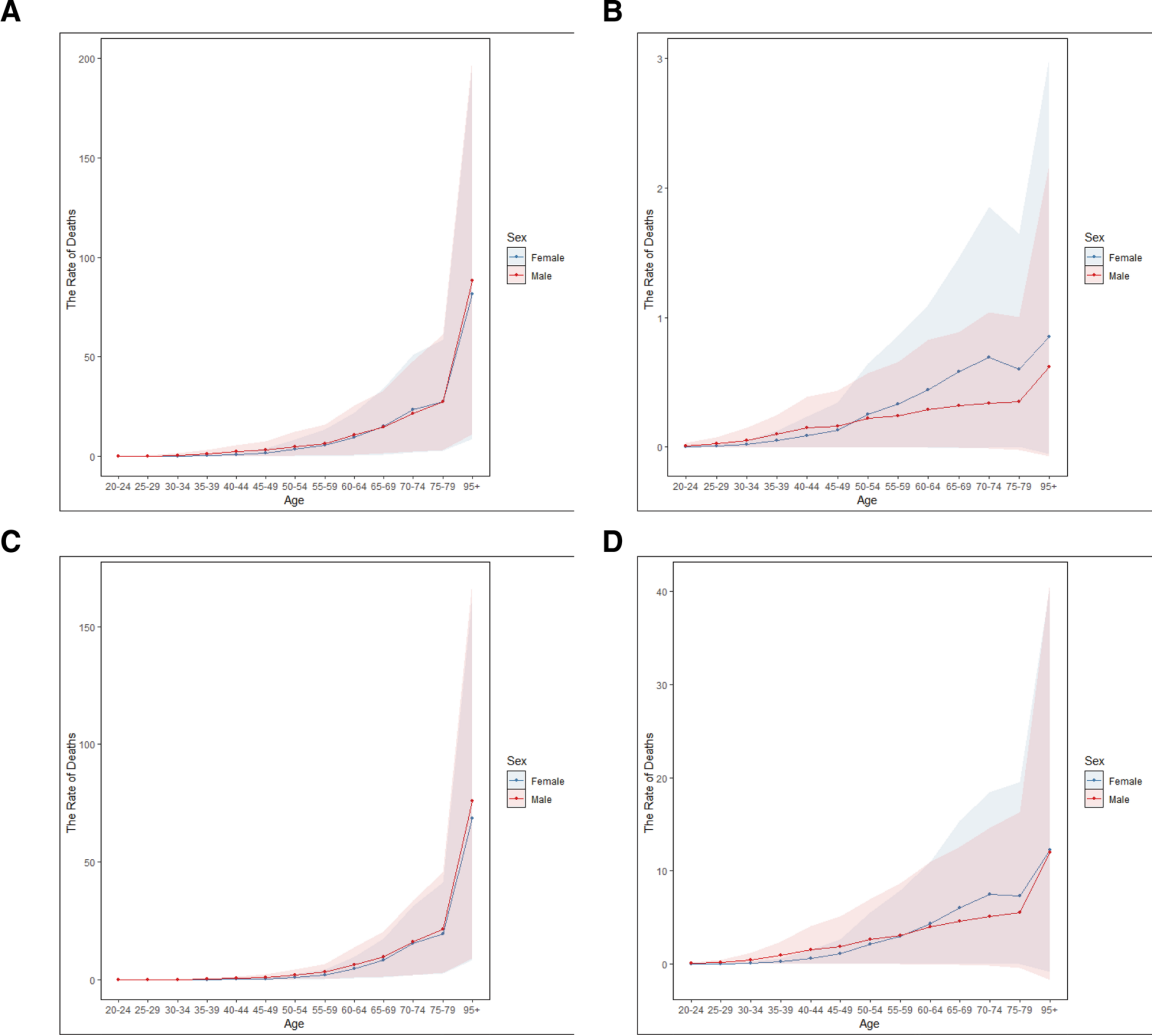
Longitudinal age curves of high BMI-attributable stroke and subtype mortality in China. Data for females and males are shown. Fitted longitudinal age-specific rates of high BMI-attributable stroke mortality (per 100,000). (A) Stroke. (B) SAH. (C) IS. (D) ICH. Y-axis: per 100,000 individuals. BMI = body mass index, ICH = intracerebral hemorrhage, IS = ischemic stroke, SAH = subarachnoid hemorrhage.

In China, there were 71,095 (95% CI: 6019–157,250) deaths in 2021 due to stroke attributable to high BMI, with 36,308 (95% CI: 3634–83,507) deaths among men and 36,308 (95% CI: 3634–83,507) deaths among women (Table 1). In 2021, ASMR was 3.47 deaths (95% CI: 0.3–7.53) per 100,000; DALY was 95.25 (95% CI: 7.96–207.77) per 100,000; years lived with disability was 14.13 (95% CI: 1.67–29.24) per 100,000; and years of life lost was 81.13 (95% CI: 6.42–179.1) per 100,000. ASMR according to stroke subtype was as follows: SAH, 0.1 deaths (95% CI: 0–0.27) per 100,000; IS, 2.21 deaths (95% CI: 0.3–4.58) per 100,000; and ICH, 1.16 deaths (95% CI: −0.01–2.94) per 100,000 (Table 1). The number of deaths from stroke (mostly IS and ICH) increased markedly between the ages of 50 and 54 years and 75 to 79 years (Fig. 1). Notably, the mortality of IS was higher in men than in women in all age groups. ICH occurred more frequently in men than in women before age 60 to 64 years, with the trend reversing at later ages.

**Table 1:** All-age cases and age-standardized deaths, DALYs, YLDs, and YLLs in 2021 for stroke attributable to high BMI in China.

Measure	All-ages cases			Age-standardized rates per 100,000 people		
	Total	Male	Female	Total	Male	Female
Stroke						
Deaths	71,095 (6019–157,250)	36,308 (3634–83,507)	34,788 (2665–76,354)	3.47 (0.3–7.53)	3.89 (0.4–8.79)	3.16 (0.24–6.89)
DALYs	2,006,701 (168,750–4379,523)	1,052,434 (94,892–2397,109)	954,267 (70,453–2103,754)	95.25 (7.96–207.77)	104.55 (9.55–237.58)	86.46 (6.4–189.87)
YLDs	294,244 (34,947–608,121)	125,011 (14,591–260,245)	169,233 (20,036–352,598)	14.13 (1.67–29.24)	12.23 (1.41–25.27)	15.95 (1.88–33)
YLLs	1,712,457 (130,667–3,798,368)	927,423 (85,279–2,157,462)	785,034 (51,844–1,778,749)	81.13 (6.42–179.1)	92.31 (8.6–215.32)	70.51 (4.65–159.54)
Subarachnoid hemorrhage						
Deaths	2206 (−16–5580)	952 (−13–2572)	1254 (1–3277)	0.1 (0–0.27)	0.1 (0–0.25)	0.11 (0–0.29)
DALYs	77,907 (−510–193,962)	35,638 (−381–92,848)	42,269 (13–103,599)	3.8 (−0.03–9.49)	3.62 (−0.04–9.18)	3.94 (0–9.68)
YLDs	10,755 (−29–26,654)	3953 (−43–10,095)	6803 (2–16,736)	0.54 (0–1.34)	0.42 (0–1.06)	0.67 (0–1.63)
YLLs	67,151 (−447–169,519)	31,685 (−338–84,456)	35,466 (9–90,143)	3.26 (−0.02–8.23)	3.21 (−0.04–8.3)	3.27 (0–8.3)
Ischemic stroke						
Deaths	44,263 (6069–91,658)	23,131 (3175–48,467)	21,132 (2905–42,490)	2.21 (0.3–4.58)	2.57 (0.35–5.39)	1.94 (0.26–3.93)
DALYs	1,188,225 (166,008–2,395,996)	620,513 (87,244–1,274,378)	567,713 (80171–1,134,837)	56.21 (7.84–113)	61.78 (8.61–126.27)	51.45 (7.25–102.18)
YLDs	249,855 (34,101–504,669)	105,084 (14,216–209,868)	144,771 (19,854–294,842)	11.91 (1.62–23.95)	10.2 (1.38–20.27)	13.55 (1.86–27.51)
YLLs	938,370 (131,104–1,936,470)	515,429 (72,289–1,100,884)	422,941 (59,314–854,332)	44.3 (6.16–91.71)	51.58 (7.18–108.74)	37.89 (5.29–77.01)
Intracerebral hemorrhage						
Deaths	44,263 (6069–91,658)	23,131 (3175–48,467)	21,132 (2905–42,490)	1.16 (−0.01–2.94)	1.22 (−0.02–3.27)	1.11 (0–2.8)
DALYs	1,188,225 (166,008–2,395,996)	620,513 (87,244–1,274,378)	567,713 (80,171–1,134,837)	35.24 (−0.29–87.45)	39.14 (−0.52–101.61)	31.07 (0.02–78.09)
YLDs	249,855 (34,101–504,669)	105,084 (14,216–209,868)	144,771 (19,854–294,842)	1.68 (−0.01–4.09)	1.62 (−0.02–4.06)	1.72 (0–4.23)
YLLs	938,370 (131,104–1,936,470)	515,429 (72,289–1,100,884)	422,941 (59,314–854,332)	33.56 (−0.28–83.49)	37.52 (−0.51–97.01)	29.34 (0.02–74.35)

BMI = body mass index, DALYs = disability-adjusted life-years, YLDs = years lived with disability, YLLs = years of life lost.

**Figure 2.**
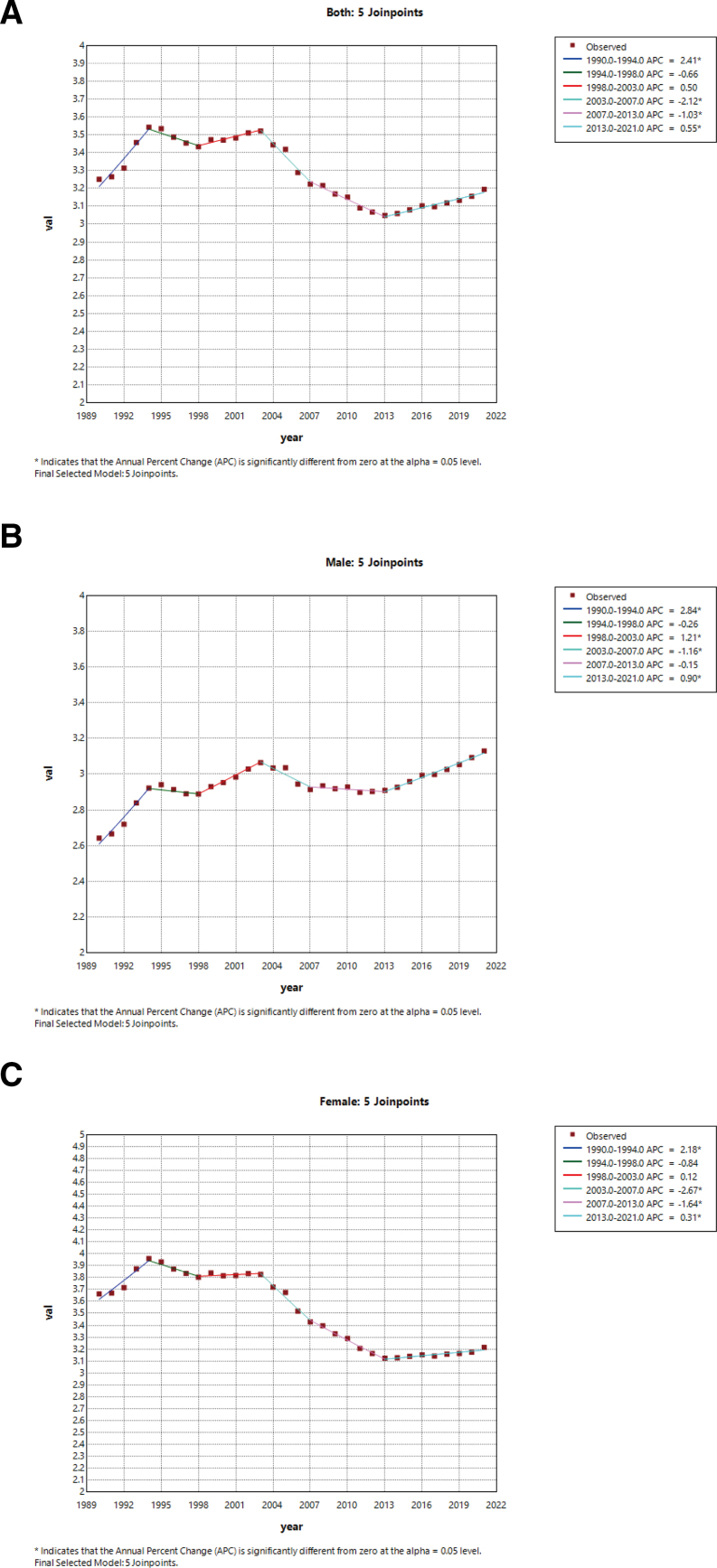
The APC of age-standardized mortality rates of stroke attributable to high BMI in global from 1990 to 2021 (* means *P*-values < .05 and significant results). (A) Both sexes. (B) Males. (C) Females. APC = annual percent change, BMI = body mass index.

**Figure 3.**
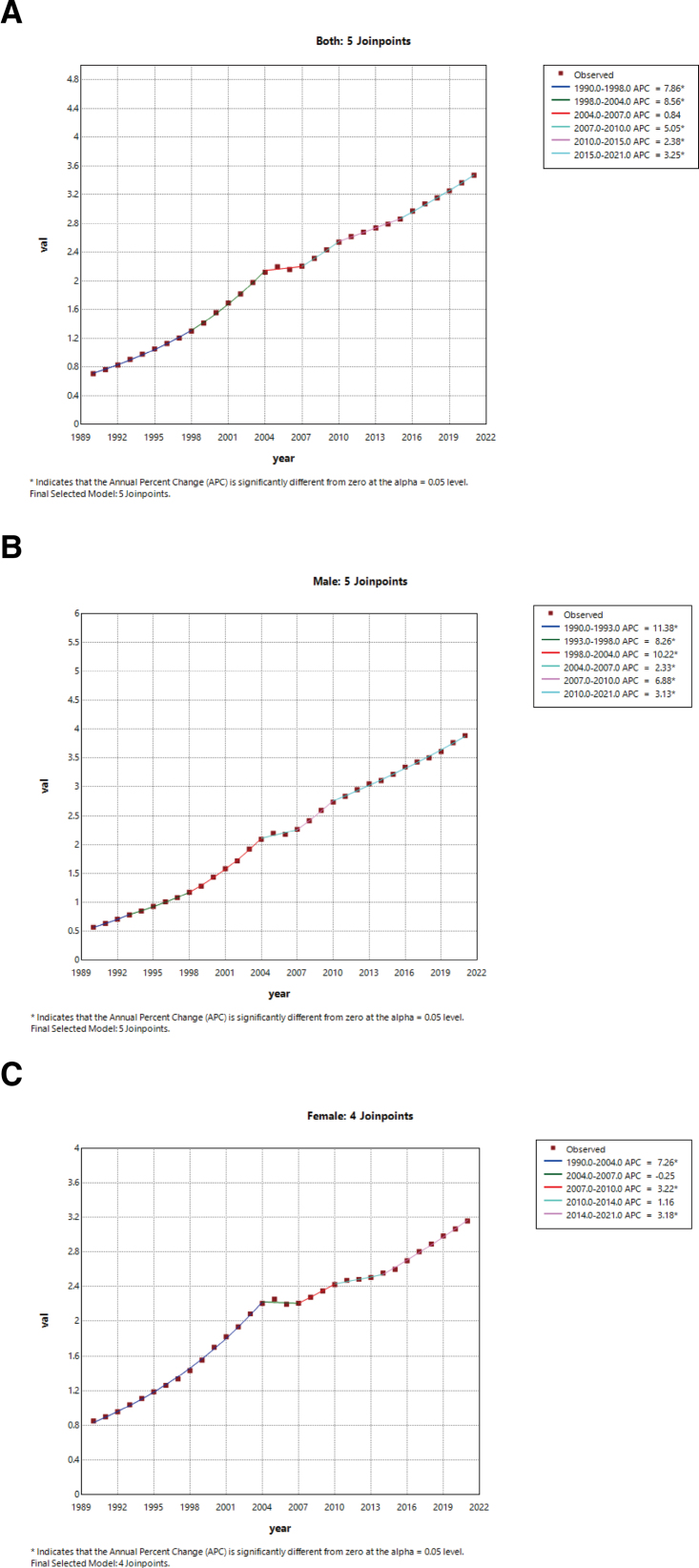
APC of age-standardized mortality rates of stroke attributable to high BMI in China (1990–2021). *Statistically significant at *P* < .05. (A) Both sexes. (B) Males. (C) Females. APC = annual percent change, BMI = body mass index.

Join-point regression analysis of ASMR of stroke attributable to high BMI in China and worldwide from 1990 to 2021 revealed that ASMRs increased before 2003 (*P* < .05) and decreased slightly after 2013 (Table S1 and Fig. 2A). A similar but attenuated trend was observed in males (Fig. 2B), while females showed sharper differences among periods (*P* < .05) (Fig. 2C). In China, the APC for ASMR in China increased steadily and significantly from 1990 to 2021 (1990–1998: APC = 7.86, *P* < .05; 2015–2021: APC = 3.25, *P* < .05; 1990–2021: AAPC = 5.23, *P* < .05) (Fig. 3A). Similar increasing trends were observed in both sexes, although men (Fig. 3B) showed a larger increase than women (Fig. 3C). The lag analyses are shown as Figure S3, including the overall cumulative effect of high BMI on stroke mortality, the exposure-lagged response relationship between high BMI and stroke mortality, and the lag effect of high BMI on stroke mortality. Figure S4 shows that the percentage of stroke attributed to high BMI in all gender groups showed a significant upward trend, with the female group generally having a higher percentage than the male group, increasing by over 300% between 1990 and 2021.

### 3.2. Projected trends in stroke mortality attributable to high BMI, 2021 to 2036

**Figure 4.**
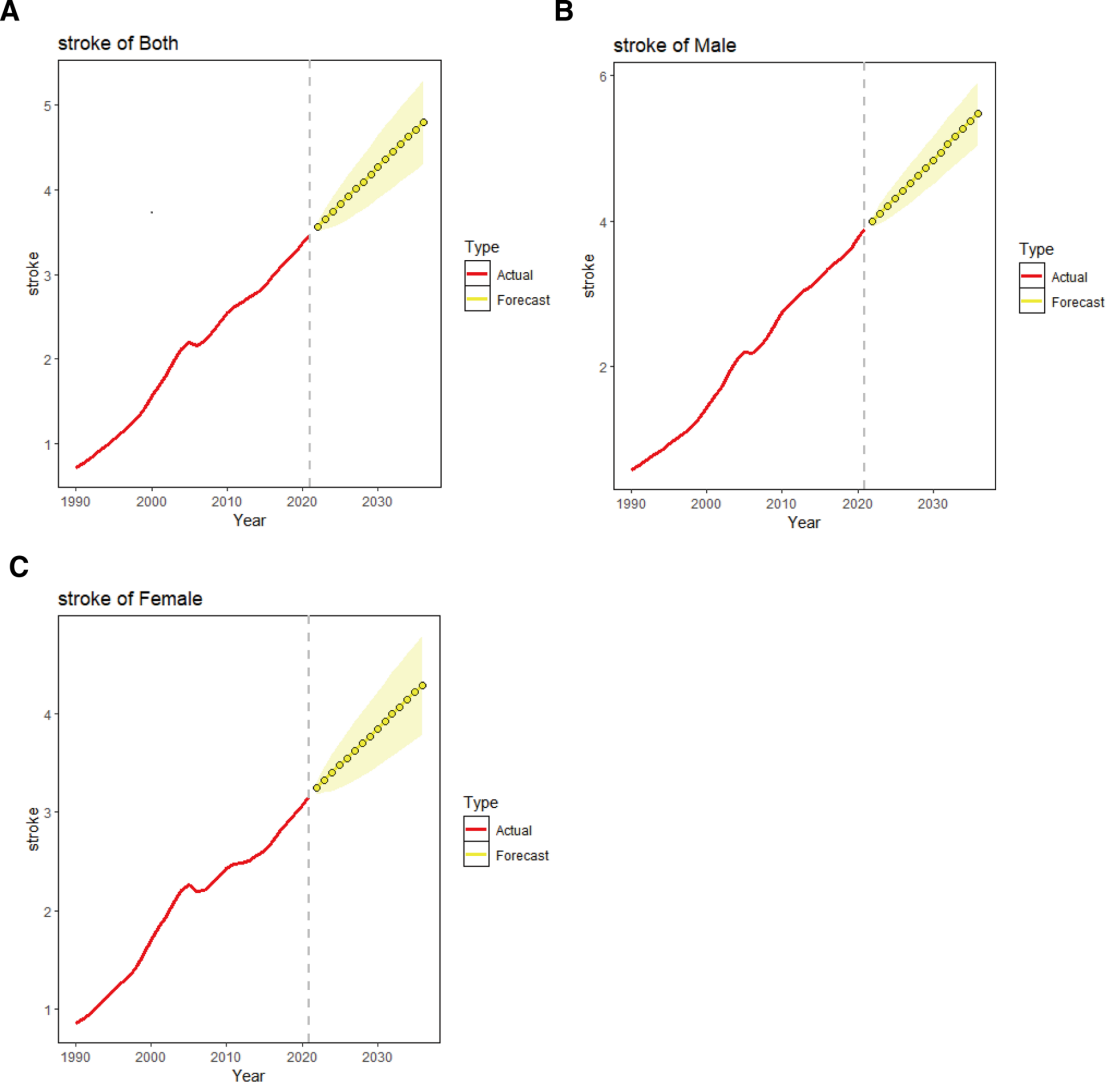
Predicted trends of stroke mortality attributable to high BMI in China over the next 15 yr (2022–2036). Red and green lines represent the actual trend of ASMRs from 1990–2021; yellow dotted lines and shaded regions represent the predicted trend and corresponding 95% CI. ASMR = age-standardized mortality rate, BMI = body mass index, CI = confidence interval.

ARIMA was used to model trends in stroke mortality attributable to high BMI over the 15-year period from 2021 to 2036. Residual diagnostics, including Q–Q plots and ACF/PACF plots (Fig. S5), suggested that the residuals approximately followed a normal distribution, while minor autocorrelation remained at specific lags. These findings indicate that the ARIMA model adequately captured the main temporal structure for descriptive forecasting, although residual dependence suggests that long-term projections should be interpreted with caution. The optimized model was selected as (0, 1, 2), with an AIC value of −125.43 after filtering with the auto.arima() function. Sex-specific ARIMA models were (0, 1, 1) for males (AIC = −111.51) and (0, 1, 2) for females (AIC = −122.53) (Fig. 4). Stroke mortality attributable to high BMI continued to increase in both sexes over the modeled time period, from 3.47 per 100,000 in 2021 to 4.80 per 100,000 in 2036 (Table S2).

### 3.3. Sensitivity analysis

A sensitivity analysis was performed. The main findings are that high BMI was significantly negatively associated with stroke mortality across all models (*P* < .001). The changes in the coefficient for high BMI after sequentially adding covariates were −30.6151 for model 1 (BMI only), −28.1613 for model 2 (+ tobacco use), and −25.9878 for model 3 (+ all covariates). Hence, the total change was 15.11%, indicating a good robustness of the association. In addition, model explanatory power increased progressively: *R*^2^ increased from 0.9585 (model 1) to 0.9952 (model 3). Hence, tobacco use was significantly positively associated with stroke mortality. Low physical activity was significantly negatively associated with stroke mortality. On the other hand, high alcohol consumption showed no significant association with stroke mortality.

## 4. Discussion

This study provides an updated overview of the large disease burden of stroke attributable to high BMI in China. Since 1990, the burden has shown an upward trend in China, whereas the global trend shows a different trajectory. In China, this burden varies by stroke subtype, gender, and age group. Age-specific analysis revealed 2 notable increases in ASMR: one between the ages of 5054 years and another between 7579 years. Across all age categories, men generally had higher rates of mortality from IS than women. However, for ICH, the mortality rates were higher among men than women before 60 to 64 years, after which the trend reversed. Meanwhile, the mortality burden of stroke attributable to high BMI in China is expected to increase from 3.47 per 100,000 in 2021 to 4.80 per 100,000 in 2036.

According to the 2020 China Nutrition and Chronic Disease Survey, the obesity rate among Chinese adults was 16.4% in 2018; rates differed between sexes (18.2% in men and 14.7% in women) and across age groups.^[22]^ A nationwide cross-sectional study of 15.8 million adults showed that the prevalence of obesity in Chinese men was highest between the ages of 35 to 39 years versus 70 to 74 years for women.^[23]^ This distribution is basically consistent with our research results. In a study of 1781 Chinese adults, a larger proportion of men compared with women were overweight or obese (defined as BMI ≥ 25 kg/m^2^) with visceral obesity (visceral fat area ≥ 100 cm^2^ at the level of the third lumbar spine based on imaging classification).^[24]^ Meanwhile, men are more likely to accumulate adipose tissue in the trunk and abdomen, whereas women are more likely to have accumulation in the buttocks and thighs.^[25,26]^ Other studies have shown that visceral fat (visceral adipose tissue and intrahepatic and intramyocellular lipids) was positively correlated with metabolic risk, whereas lower limb fat was negatively correlated with cardiometabolic risk.^[12,27,28]^ Because of their fat distribution, men have a higher metabolic risk than women. In addition, due to differences in sex hormone levels between men and women^[29]^: subcutaneous adipose tissue has a higher concentration of estrogen/progesterone receptors than androgen receptors, whereas visceral adipose tissue has a higher concentration of androgen receptors. Moreover, 4 key atherogenic lipids (low-density lipoproteins, very low-density lipoproteins, triglycerides, and apolipoproteins) were detected at higher levels in men with obesity than in women with obesity.^[30–32]^ Sex hormones also impact eating behaviors, including homeostatic and hedonic eating: 2 cross-sectional studies with large sample sizes found that women preferred high-carbohydrate foods, whereas men preferred fat-rich foods.^[33,34]^ Additionally, women generally place greater importance on weight management than men and may be more likely to seek out medical or surgical treatments for weight control.^[33,34]^ In conclusion, sex and age differences in stroke disease burden due to high BMI may be related to individual differences in lifestyle, hormonal, genetic, socio-political, cultural, and environmental factors.

Compared with the recent global assessment,^[35]^ which highlights a declining age-standardized IS burden attributable to high BMI despite rising absolute case numbers, the present findings indicate a sustained and projected increase in high BMI-related stroke mortality in China, encompassing ischemic and hemorrhagic subtypes and demonstrating marked heterogeneity by age and sex. These divergent trajectories suggest that China lies at the nexus of a global shift of high BMI-attributable stroke burden toward middle-SDI settings, while still experiencing worsening mortality rates, underscoring the need for aggressive, context-specific metabolic risk reduction and stroke prevention strategies.

Indeed, with the economic transformation of China, changes in dietary patterns, lifestyles and population structure have led to the expansion of the population with increased BMI and the increase in the risk of stroke: the westernization of dietary structure and the increase in the intake of animal fat and processed food^[36]^; the cycle of mental health and metabolic disorders, the vicious cycle of stress-diet, forming “stress obesity”^[37]^; Uneven distribution of medical resources, low health literacy in rural areas, and insufficient coverage of stroke first aid network^[38,39]^; The synergistic effect of metabolic syndrome, and the additive effect of hypertension, diabetes and high BMI were not effectively controlled^[40]^; Aging combined with metabolic diseases increases the risk of stroke death.^[41,42]^ The increasing trend of high BMI-related stroke mortality in China not only epitoses the chronic disease burden of globalization, but also reflects the special challenges of localized social transformation. It is necessary to implement the whole chain of “prevention-treatment-rehabilitation” intervention to reverse this trend, focusing on male high-risk groups and rural areas, and paying attention to the synergistic effect of aging and metabolic diseases. Projections for 2036 warn of the health equity gap that is likely to widen if we rely solely on technological improvements and ignore social determinants. More disruptive thinking, such as incorporating health impact assessment into all macroeconomic policymaking processes, is needed to overcome the challenges of population aging and metabolic crisis.

However, some studies have shown that higher BMI is associated with reduced mortality, improved functional outcomes, and improved self-reported health after 90 days.^[43,44]^ This phenomenon is known and displays as a nonlinear association between body weight and stroke outcome. In patients with ICH, the risk of disability showed a U-shaped association with BMI.^[45]^ In chronic stroke survivors, the nonlinear association between body weight and stroke outcome is thought to be related to the negative effect of low BMI on muscle mass and intramuscular fat.^[46]^ This may be related to the following: normal weight, overweight, or obese individuals have greater nutritional reserves than underweight individuals; At the same time, underweight or severely obese individuals may have limited ability to recover from stroke, thereby increasing their risk of disability; Subnormal BMI could also be due to chronic medical conditions or the possible co-occurrence of smoking and obesity with other medical complications, which would lead to worse outcomes after stroke. It is therefore critical to increase public health awareness and the importance of maintaining a normal BMI, increasing muscle mass, and decreasing muscle fat to reduce the risk of stroke.

The present study identified a higher stroke mortality rate among men, but the mechanistic explanation attributing differences to fat distribution alone is insufficient. MR research has revealed that visceral adiposity is associated with elevated release of pro-inflammatory cytokines, such as IL-6 and TNF-α, which accelerate systemic and vascular inflammation, which are processes shown to causally increase stroke risk. MR analyses confirm that higher genetically determined BMI significantly raises the risk of IS (relative risks from 1.08 to 1.25 per standard deviation increase in BMI), while waist-to-hip ratio and fat mass indices, representing visceral fat distribution, are also independently associated with cerebrovascular disease risk.^[47–50]^ This new genetic evidence calls for a more nuanced interpretation beyond traditional anthropometric measurements and underscores the necessity of integrating molecular mechanisms, particularly those mediated by inflammatory cytokines, into discussions of sex-specific risk differences. Additionally, potential epigenetic effects, such as those stemming from maternal BMI, should not be disregarded, as MR studies and related analyses point to plausible transgenerational influences on metabolic traits and vascular risk in offspring.^[51]^ Regarding conclusions, an important over-extrapolation is evident when increases in mortality attributable to high BMI are equated directly with increases in disease prevalence. Advances in acute intervention, such as thrombolysis and neurointerventional strategies, can reduce mortality even amid persistent (or increasing) prevalence of high BMI. It is therefore critical to distinguish between drivers of disease burden (e.g., rising BMI prevalence) and factors improving outcomes (e.g., newer therapies), to avoid confounding incidence and survival dynamics.^[47,52]^ Causality is strengthened by MR studies, which, as in recent meta-analyses, demonstrate the direct effect of BMI on stroke risk independent of classic confounding, and even more importantly, on post-stroke functional recovery. For example, higher genetically predicted BMI causally associates with worse motor, cognitive, and global recovery after IS (odds ratio for improved motor outcome 0.37 per 1-SD increase in BMI). These findings highlight not only increased risk for incident stroke but also poorer outcomes thereafter, which should inform both prevention and rehabilitation frameworks.^[52]^ Future research should continue to leverage genetically informed designs to untangle these causal pathways, incorporate molecular and epigenetic mechanisms, and clearly differentiate between contributors to disease burden and factors mitigating adverse outcomes. By grounding the interpretation in both mechanistic evidence and robust causal inference, the results can more accurately capture the complexity of obesity-stroke relationships.

This study had several limitations that should be noted. As the data source was the GBD study, our analyses relied on the accuracy and validity of the methodological and statistical modeling approaches used by the GBD investigators. The availability and quality of data inputs may have also impacted the results, although the GBD analysis applied rigorous methods to address missing information. Finally, we were unable to explore differences between more fine-grained subgroups of individual characteristics that may show different patterns.

## 5. Conclusion

This study provides the latest evidence supporting the long-term increasing trend in stroke burden attributable to high BMI in China. The burden varies by subtypes, gender, and age groups. Furthermore, the mortality burden of stroke attributable to high BMI is projected to increase from 3.47 per 100,000 in 2021 to 4.80 per 100,000 in 2036. Therefore, China’s public health authorities should focus on high-risk groups and ensure the implementation of a comprehensive “prevention-treatment-rehabilitation” intervention to reverse this trend.

## Acknowledgments

We thank Charlesworth Author Services for English language editing.

## Author contributions

**Data curation:** Ye Tian.

**Formal analysis:** Wenbin Tian, Pei Zhang, Ning Yu, Chao Liu, Xuefang Liu.

**Methodology:** Ye Tian, Huaihai Lu.

**Writing – original draft:** Ye Tian.

**Writing – review & editing:** Huaihai Lu.


