# Supplementary material for: Stroke burden attributable to high body mass index in China from 1990 to 2021 and projections to 2036 based on global burden of disease data
Source: Medicine (Baltimore). 2026 May 29;105(22):e49042. doi: 10.1097/MD.0000000000049042 (PMC13225537; doi:10.1097/MD.0000000000049042)
Supplement: Supplementary file 1 [file medi-105-e49042-s001.pdf]

Table S1. Trends of age-standardized mortality rates of stroke attributable to high body-mass index from 1990 to 2021 in global and China using join-point regression model

	Both sexes				Men				Women			
	**Segment**	**Period**	**APC (95% CI)**	**P value**	**Segment**	**Period**	**APC (95% CI)**	**P value**	**Segment**	**Period**	**APC (95% CI)**	**P value**
	1	1990-1994	2.41 (1.77 - 3.06)	<0.001	1	1990-1994	2.84 (2.24 - 3.45)	<0.001	1	1990-1994	2.18 (1.50 - 2.86)	<0.001
	2	1994-1998	-0.66 (-1.66 - 0.35)	0.182	2	1994-1998	-0.26 (-1.17 - 0.67)	0.559	2	1994-1998	-0.84 (-1.89 - 0.21)	0.109
	3	1998-2003	0.50 (-0.14 - 1.14)	0.114	3	1998-2003	1.21 (0.61 - 1.80)	<0.001	3	1998-2003	0.12 (-0.55 - 0.79)	0.702
	4	2003-2007	-2.12 (-3.09 - -1.13)	<0.001	4	2003-2007	-1.16 (-2.07 - -0.24)	0.017	4	2003-2007	-2.67 (-3.69 - -1.63)	<0.001
	5	2007-2013	-1.03 (-1.48 - -0.59)	<0.001	5	2007-2013	-0.15 (-0.56 - 0.27)	0.459	5	2007-2013	-1.64 (-2.11 - -1.18)	<0.001
	6	2013-2021	0.55 (0.32 - 0.77)	<0.001	6	2013-2021	0.90 (0.69 - 1.11)	<0.001	6	2013-2021	0.31 (0.08 - 0.53)	0.012
**Global**	AAPC	1990-2021	-0.03 (-0.26 - 0.20)	0.777	AAPC	1990-2021	0.58 (0.36 - 0.79)	<0.001	AAPC	1990-2021	-0.40 (-0.64 - -0.16)	0.001
	1	1990-1998	7.86 (7.57 - 8.15)	<0.001	1	1990-1993	11.38 (9.26 - 13.55)	<0.001	1	1990-2004	7.26 (7.11 - 7.42)	<0.001
	2	1998-2004	8.56 (8.11 - 9.00)	<0.001	2	1993-1998	8.26 (7.33 - 9.20)	<0.001	2	2004-2007	-0.25 (-2.67 - 2.23)	0.832
	3	2004-2007	0.84 (-0.84 - 2.55)	0.305	3	1998-2004	10.22 (9.69 - 10.75)	<0.001	3	2007-2010	3.22 (0.73 - 5.77)	0.014
	4	2007-2010	5.05 (3.33 - 6.79)	<0.001	4	2004-2007	2.33 (0.40 - 4.30)	0.021	4	2010-2014	1.16 (-0.03 - 2.35)	0.055
	5	2010-2015	2.38 (1.88 - 2.89)	<0.001	5	2007-2010	6.88 (4.87 - 8.94)	<0.001	5	2014-2021	3.18 (2.84 - 3.51)	<0.001
	6	2015-2021	3.25 (2.97 - 3.52)	<0.001	6	2010-2021	3.13 (3.00 - 3.25)	<0.001	…	…	…	…
**China**	AAPC	1990-2021	5.23 (4.97 - 5.49)	<0.001	AAPC	1990-2021	6.36 (6.00 - 6.72)	<0.001	AAPC	1990-2021	4.40 (4.03 - 4.77)	<0.001
